# Improved cryopreservation of in vitro produced bovine embryos using FGF2, LIF, and IGF1

**DOI:** 10.1371/journal.pone.0243727

**Published:** 2021-02-03

**Authors:** Katy S. Stoecklein, M. Sofia Ortega, Lee D. Spate, Clifton N. Murphy, Randall S. Prather

**Affiliations:** Division of Animal Sciences, University of Missouri, Columbia, Missouri, United States of America; School of Sciences and Languages, Sao Paulo State University (UNESP), BRAZIL

## Abstract

In vitro embryo production systems are limited by their inability to consistently produce embryos with the competency to develop to the blastocyst stage, survive cryopreservation, and establish a pregnancy. Previous work identified a combination of three cytokines [fibroblast growth factor 2 (FGF2), leukemia inhibitory factor (LIF), and insulin-like growth factor 1 (IGF1)], called FLI, that we hypothesize improve preimplantation development of bovine embryos in vitro. To test this hypothesis, FLI was supplemented into oocyte maturation or embryo culture medium. Embryos were produced in vitro using abattoir-derived oocytes and fertilized with sperm from a single bull known to have high fertility. After an 18–20 h fertilization period, putative zygotes were cultured in synthetic oviductal fluid (SOF) for 8 days. The addition of FLI to the oocyte maturation medium increased (*P* < 0.05) the dissociation of transzonal projections at 12, 18, and 24 h of maturation, as well as, the proportion of oocytes that reached the metaphase II stage of meiosis. Additionally, lipid content was decreased (*P* < 0.05) in the blastocyst stage embryo. The addition of FLI during the culture period increased development to the blastocyst stage, cytoskeleton integrity, and survival following slow freezing, as well as, decreased post thaw cell apoptosis (*P* < 0.05). In conclusion, the supplementation of these cytokines in vitro has the potential to alleviate some of the challenges associated with the cryo-survival of in vitro produced bovine embryos through improving embryo development and embryo quality.

## Introduction

In vitro embryo production is an assisted reproductive technique used in livestock production to maximize the use of valuable genetics [1]. Of the livestock species, *Bos taurus* heavily rely on these technologies compared to others. In 2018, a total of 1,499,367 transferrable embryos were produced worldwide, from which 68.7% were in vitro produced (IVP) [2]. Though the industry is trending toward IVP embryos, the majority (73.2%), are transferred fresh; in part due to the reduced ability of IVP embryos to withstand cryopreservation as compared to embryos produced in vivo [3–5]. Therefore, it is important to understand the IVP system to increase viable embryo production in vitro as well as survival after cryopreservation.

Currently, less than 50% of the IVP bovine embryos will develop to the blastocyst stage. One of the main causes of this low yield has been attributed to inadequate oocyte cytoplasmic and nuclear maturation [6, 7]. A second critical period is culture after fertilization. There is evidence that the environment of the embryo can affect the transcript abundance for genes important for preimplantation development [e.g. *GJA1*, *BAX*, Interferon-tau *(IFNT*)], increase lipid content, reduce numbers of mitochondria, and alter the cytoplasmic:nuclear ratio [8, 9]. One well-reported characteristic of IVP embryos is their increased susceptibility to cryopreservation, likely due to the variation in blastocyst quality observed in IVP embryos [9].

Quality parameters that define the competency of an oocyte or embryo to survive cryopreservation and establish a pregnancy are not well defined. In the oocyte, transzonal projections have been posed as indicators of oocyte quality because they serve as bidirectional communication channels between the oocyte and surrounding cumulus cells [10, 11]. Previous work has shown that transzonal projections may facilitate fatty acid binding protein transport from cumulus cells into the oocyte, and abnormal dissociation of the projections could lead to increased lipid accumulation and metabolic dysfunction [12].

In the embryo, cell number and allocation of cells within the blastocyst (inner cell mass and trophectoderm) have been suggested as indicators of embryo developmental potential [13–15]. However, embryos that appeared cleaved and developed to the blastocyst stage quicker than their counterparts did not have an increased number of cells in the inner cell mass [16]. Another indicator of embryo quality or competence is the accumulation of reactive oxygen species, which can be indicators of cellular stress and altered metabolism [17–19]. Furthermore, cellular integrity, determined by cytoskeleton analysis and cell organization, is suggested to be an indicator of embryo quality in other species [20, 21]. It is evident that there is a need to more clearly define the contribution of these parameters to embryo quality, specifically during in vitro production where embryo viability appears to be compromised [22–25].

One approach to increase the efficiency of the IVP system would be the addition of embryokines that are present in the female reproductive tract following ovulation [26]. The addition of molecules such as LIF or FGF2 to maturation or IGF1 or IL6 to culture medium have improved the embryo’s ability to develop to the blastocyst stage [27, 28], increase cell number [29], survive cryopreservation [30–32], and resist heat stress/shock [33]. Furthermore, certain cytokine combinations such as, LIF and IGF-1, IGF-I, IGF-II, bFGF, TGF-β1, GM-CSF, and LIF, or EGF and IGF-1 have acted additively to improve in vitro embryo production through an increase in embryo development and/or quality [34–36]. Three cytokines, FGF2 (40 ng/ml), LIF (20 ng/ml), and IGF1 (20 ng/ml) (when combined termed FLI), were identified for their potential roles as candidates to improve embryonic development in vitro in the pig [37]. In cattle, FGF2 and LIF are involved in pluripotency maintenance, stimulation of *IFNT* production [38], and cryotolerance [30, 31], while IGF1 is thought to increase cell survival in response to stress [33]. Thus, we hypothesize, that these three cytokines have the potential to increase oocyte maturation and embryonic development to the blastocyst stage as well as oocyte and embryo quality when added to oocyte maturation medium or embryo culture medium.

## Materials and methods

### In vitro embryo production media

All embryo production media: Oocyte maturation (OMM), fertilization (IVF-TALP), wash (HEPES-TALP), culture (SOF-BE2), and the supplement penicillamine, hypotaurine, and epinephrine (PHE) were prepared in house unless otherwise noted following recipes previously published [39]. Sperm purification gradient Isolate® (Irvine Scientific, Santa Ana, CA, USA) was acquired ready to use. FLI was supplemented at the dosage of FGF2 (40ng/ml), LIF (20ng/ml), and IGF1 (20ng/ml) [37] to the maturation medium or culture medium.

### FLI supplementation during oocyte maturation

**Fig 1:**
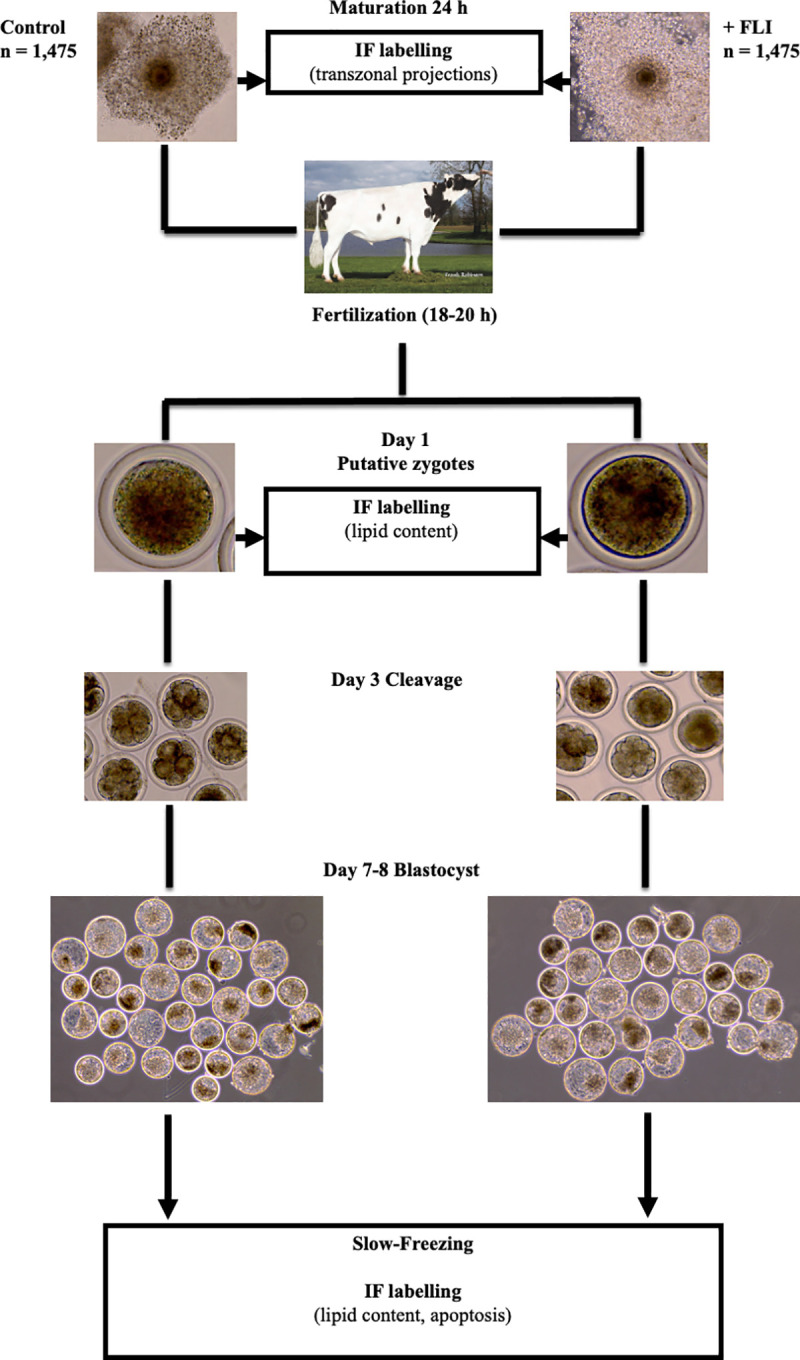
Experimental setup of FLI supplemented to oocyte maturation medium. Cumulus oocyte complexes (COCs) were matured in oocyte maturation medium (OMM) with (n = 1475) or without (n = 1475) FLI. FLI was supplemented at the dosage of FGF2 (40ng/ml), LIF (20ng/ml), and IGF1 (20ng/ml). For analysis of transzonal projections, COCs were collected at 6 h, 12 h, 18 h, 24 h after placement into OMM. At 24 h, maturation stage was recorded. Following fertilization, a subset of putative zygotes (day 1) was collected for lipid content quantification. Cleavage (at least one cellular division) was recorded on day 3 and development to the blastocyst stage was recorded on days 7 and 8. On day 7 or 8, blastocyst stage embryos were either collected for quantification of lipid content or slow frozen, thawed, and subjected to a TUNEL assay to analyze apoptosis. IF = immunofluorescent labeling.

The experimental design is presented in Fig 1.

#### Detection of transzonal projections

The number of transzonal projections in cumulus oocyte complexes (COCs) was determined by immunolocalization of F-actin filaments that connect the oocyte and cumulus cells using confocal microscopy. COCs (n = 198), with at least 2 layers of cumulus cells, and dark homogenous ooplasm [40] were matured in OMM [41] supplemented with (n = 98) or without FLI (n = 100), and collected at 6, 12, 18, and 24 h after placement into OMM. At the moment of collection, COCs were washed with phosphate buffered saline (PBS) containing 0.1% polyvinylpyrrolidone (PVP) and fixed for 20 min in droplets of PBS-PVP containing 4% paraformaldehyde. Fixed COCs were permeabilized (0.5%Triton X-100 diluted in PBS) for 30 min at room temperature, washed three times with PBS-PVP, and placed in Rhodamine phalloidin (Cytoskeleton Inc., Denver, CO, USA) diluted at 0.04 μg/ml [37]. Incubation proceeded in the dark for 45 min at room temperature. COCs were washed again three times with PBS-PVP and incubated in Hoechst 33342 (Invitrogen Molecular Probes, Waltham, MA, USA) at 1 μg/ml for 20 min in the dark. Finally, COCs were mounted on slides in Slow fade® Gold antifade reagent (Thermo Fisher Scientific, Waltham, MA, USA) and analyzed by using confocal microscopy (Leica TCS SP8 MP). Excitation/emission was 350/461 for Hoechst 33342 and 540/585 for Rhodamine phalloidin. ImageJ Software 1.46r (National Institutes of Health, Bethesda, MD, USA) was used to count the number of intact transzonal projection filaments present, which was defined by an obvious connection from the cumulus cells to the oocyte through the zona pellucida. Disrupted transzonal projections, identified by obvious breaks in the connection, were not counted.

#### Nuclear staining

In a second experiment, following maturation, oocytes [n = 304, 148 controls and 156 with FLI, across 3 replicates] were denuded and fixed with 4% paraformaldehyde. Meiotic stage was determined by nuclear labeling as previously described [42, 43]. Briefly, oocytes were permeabilized in 0.1% Triton X-100 for 30 min at room temperature, washed three times with PBS-PVP and incubated in Hoechst 33342 (1μg/ml) for 20 min in the dark. Following staining with Hoechst, oocytes were mounted on slides in Slow fade® Gold antifade reagent (Thermo Fisher Scientific) and analyzed using fluorescence microscopy. Excitation/emission was 350/461 for Hoechst 33342. The proportion of oocytes in each meiotic stage [germinal vesicle (GV), GV breakdown, metaphase 1, anaphase 1, telophase 1, and metaphase II] was recorded.

#### In vitro embryo production

For all experiments, abattoir derived COCs (n = 2,950, across 12 replicates) were received from a single company that provides abattoir derived COCs. COCs were placed in equilibrated OMM (50 COCs/tube) overlaid with oil and shipped via portable incubator set to 38.5˚C and left in tubes until collection or fertilization. OMM was supplemented with (n = 1475) or without (n = 1475) FLI. Only COCs surrounded by multiple layers of cumulus cells and with homogenous ooplasm were used. For each replicate, up to 300 mature COCs were washed three times in HEPES-TALP and placed in a dish containing 1.7 ml of IVF-TALP. The COCs were inseminated with sperm from a single Holstein bull known to have high fertility in vitro. Sperm were purified from frozen-thawed straws by using isolate gradient, washed two times by centrifugation at 100×g with HEPES-TALP and diluted in IVF-TALP to achieve a final concentration of 1 × 10^6^/ml in the fertilization dish. To improve sperm motility and capacitation, 80 μl PHE was added to the fertilization plate [44].

Fertilization proceeded for 18–20 h in a humidified atmosphere of 5% (v/v) CO_2_. At the end of fertilization, putative zygotes (oocytes exposed to sperm) were denuded from cumulus cells by vortexing for five min in 200 μl HEPES-TALP. Embryos were then washed three times in HEPES-TALP and up to 50 putative zygotes were placed in a 4-well dish containing 500 μl of SOF-BE2 overlaid with 300 μl mineral oil. Embryos were cultured at 38.5°C in a humidified atmosphere of 5% (v/v) O_2_ and 5% (v/v) CO_2_ with the balance N_2_ until collection. Cleavage of the embryos was recorded on day 3 and development to the blastocyst stage was recorded on days 7 and 8 post insemination.

#### Cryopreservation of embryos by slow-freezing

On day 7–8 post insemination, blastocysts were collected, grouped according to their stage and quality grade using IETS standards [45]. Blastocyst stage embryos that were quality 1 and stage 6 or 7 were washed three times in holding medium (HEPES-TALP, 10% FBS, 0.05% DTT) [46, 47] equilibrated to 38°C, to remove residual culture medium. Next, embryos were placed in a drop of freezing medium (BioLife Freeze Medium Ethylene Glycol with Sucrose, AgTech Inc, Manhattan, KS, USA), and allowed to equilibrate by sinking to the bottom of the dish. Straws were loaded by adding a column of freezing medium, followed by 2–3 mm of air, another column of freezing medium containing two to five embryos of the same stage and quality grade, 2–3 mm of air, and a final column of freezing medium. Loaded straws were maintained at room temperature for 5 to 15 min, then placed in the freezing machine (Cryogenic L854 Temperature Controller) equilibrated to -6°C. After 2 min, the individual straws were seeded by placing two cotton swabs in liquid nitrogen, then on each side of the top column of freezing medium until ice crystals formed. Loaded straws were held at -6°C for 10 min, and then subjected to a temperature gradient of -0.5°C/min until reaching -32°C and kept at this temperature for 10 min [32, 48]. Immediately after, straws were immersed in liquid nitrogen and stored until thawed for further analysis.

To thaw, straws were removed from liquid nitrogen, held at room temperature for 3 sec, then placed in a cryobath set at 30°C for 45 sec. Embryos were removed from straws and washed in holding medium three times. After washing, embryos were grouped according to their treatment and placed in culture plates containing SOF-BE2, with no more than 10 embryos per well. As previously described, 10% FBS was added to each well [32, 46, 49]. Hatching from the zona pellucida in vitro was used to determine cryo-survival. A total of 148 embryos (n = 73 control, n = 75 +FLI) across 5 replicates were evaluated at 24, 48, and 72 h post thaw for cryo-survival.

#### TUNEL assay

DNA fragmentation in blastocysts that re-expanded by 72 h post-thawing was determined using a terminal deoxynucleotidyl transferase dUTP nick end labeling (TUNEL) reaction kit assay (*In Situ* Cell Death Detection Kit with Fluorescein, Millipore Sigma, Burlington, MA, USA). At 72 h post thawing, expanded and hatched blastocysts were collected, washed with PBS-PVP, fixed, and permeabilized as described above. Samples were then separated into three treatments: positive control (DNAse and TUNEL solution), negative control (label solution), and the TUNEL treatment (label solution and enzyme) and subjected to the TUNEL reaction. Briefly, embryos were placed in 25 μl drops of their respective solutions in a four-well plate with water around the wells to allow for a humidified environment. Embryos were placed in a warm incubator for one h, washed in PBS-PVP, then incubated in Hoechst 33342 (1 μg/ml) for 20 min in the dark. Samples were mounted on slides in Slow fade® Gold antifade reagent (Thermo Fisher Scientific) and analyzed by fluorescence microscopy. Excitation/emission was 350/461 for Hoechst 33342 and 520/570 for TUNEL positive cells. ImageJ Software 1.46r (National Institutes of Health) was used to determine the total number of nuclei as well as the number of TUNEL positive (fragmented DNA) nuclei in each embryo (n = 36 controls, n = 38 +FLI, across 5 replicates).

#### Detection of lipid content

Lipid content in embryos was determined as previously described [50]. Briefly, putative zygotes (day 1) and expanded blastocyst (days 7 and 8) were collected and washed three times in PBS-PVP. At least two embryos were set aside for negative controls, and the remaining embryos were fixed as described before. Negative control embryos were incubated in 100 μl of 100% ethanol for 30 min to dissolve the lipids present. Following fixation, the remaining embryos were placed into a droplet of Nile Red Solution (Invitrogen Molecular Probes) for 30 min (1 μg/ml dilution). After incubation, embryos were washed with PBS-PVP three times and then nuclei were stained with Hoechst 33342 (1 μg/ml dilution) for 20 min in the dark. Samples were mounted on slides in Slow fade® Gold antifade reagent (Thermo Fisher Scientific) and analyzed by using fluorescence microscopy. Excitation/emission was 350/461 for Hoechst 33342 and 552/636 for Nile Red. ImageJ Software 1.46r (National Institutes of Health) was used to quantify mean intensity units per embryo to estimate the amount of lipids. A total of 230 zygotes (109 control, 120 +FLI) and 223 blastocyst stage embryos (116 control, 106 +FLI) were analyzed across 4 replicates in each experiment.

### FLI supplementation during embryo culture

**Fig 2:**
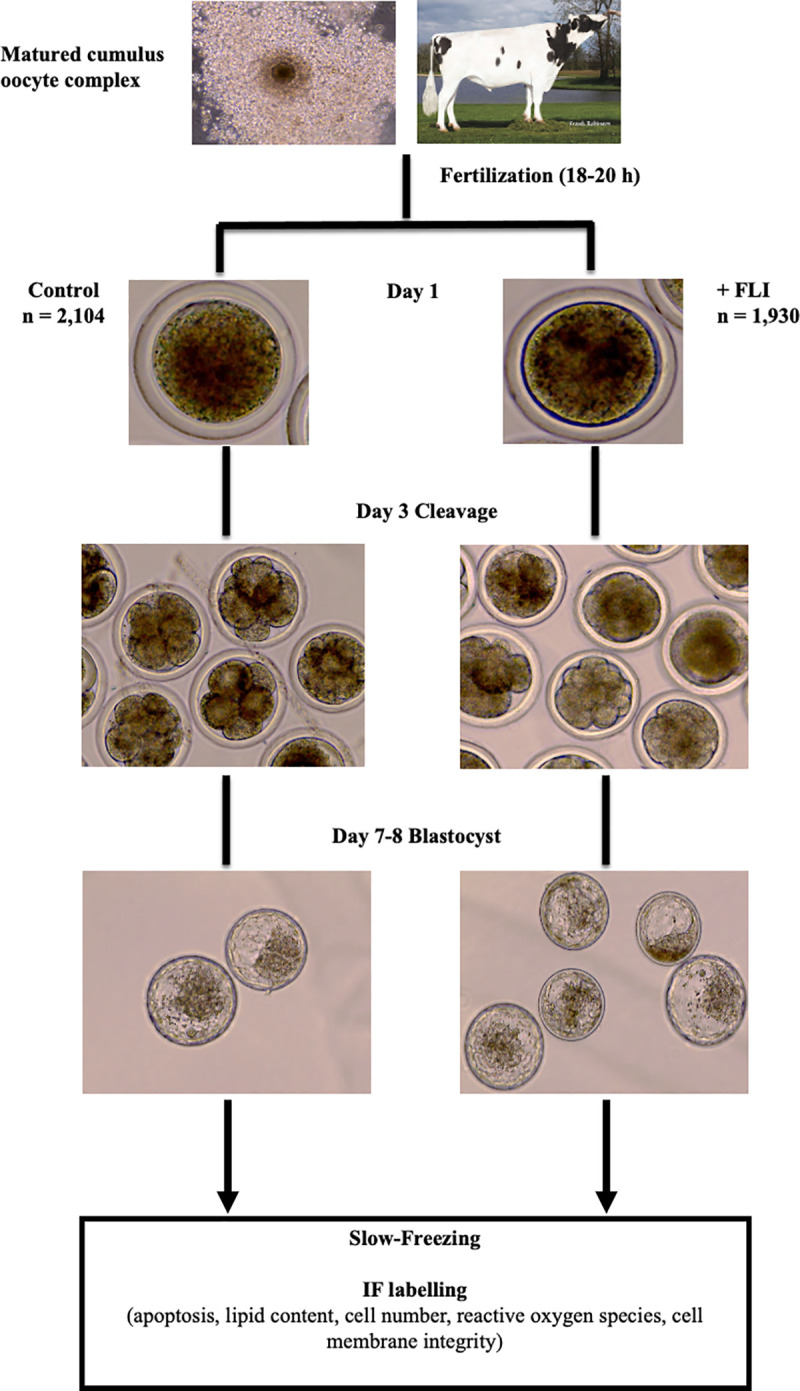
Experimental setup of FLI supplemented to embryo culture medium. Cumulus oocyte complexes (COCs) were matured in oocyte maturation medium (OMM). Following fertilization, putative zygotes were placed in culture with (n = 1,930) or without (n = 2,104) FLI. FLI was supplemented at the dosage of FGF2 (40ng/ml), LIF (20ng/ml), and IGF1 (20ng/ml). Cleavage (at least one cellular division) was recorded on day 3 and development to the blastocyst stage was recorded on day 7 and 8. Day 7 or 8, blastocyst stage embryos were either collected for cell counting, quantification of lipid content, membrane integrity analysis, quantification of reactive oxygen species, or slow frozen, and subjected to TUNEL assay to analyze apoptosis, membrane integrity analysis, or quantification of reactive oxygen species post-thaw. IF = immunofluorescent labeling.

The experimental design is presented in Fig 2.

#### In vitro embryo production

**Fig 3:**
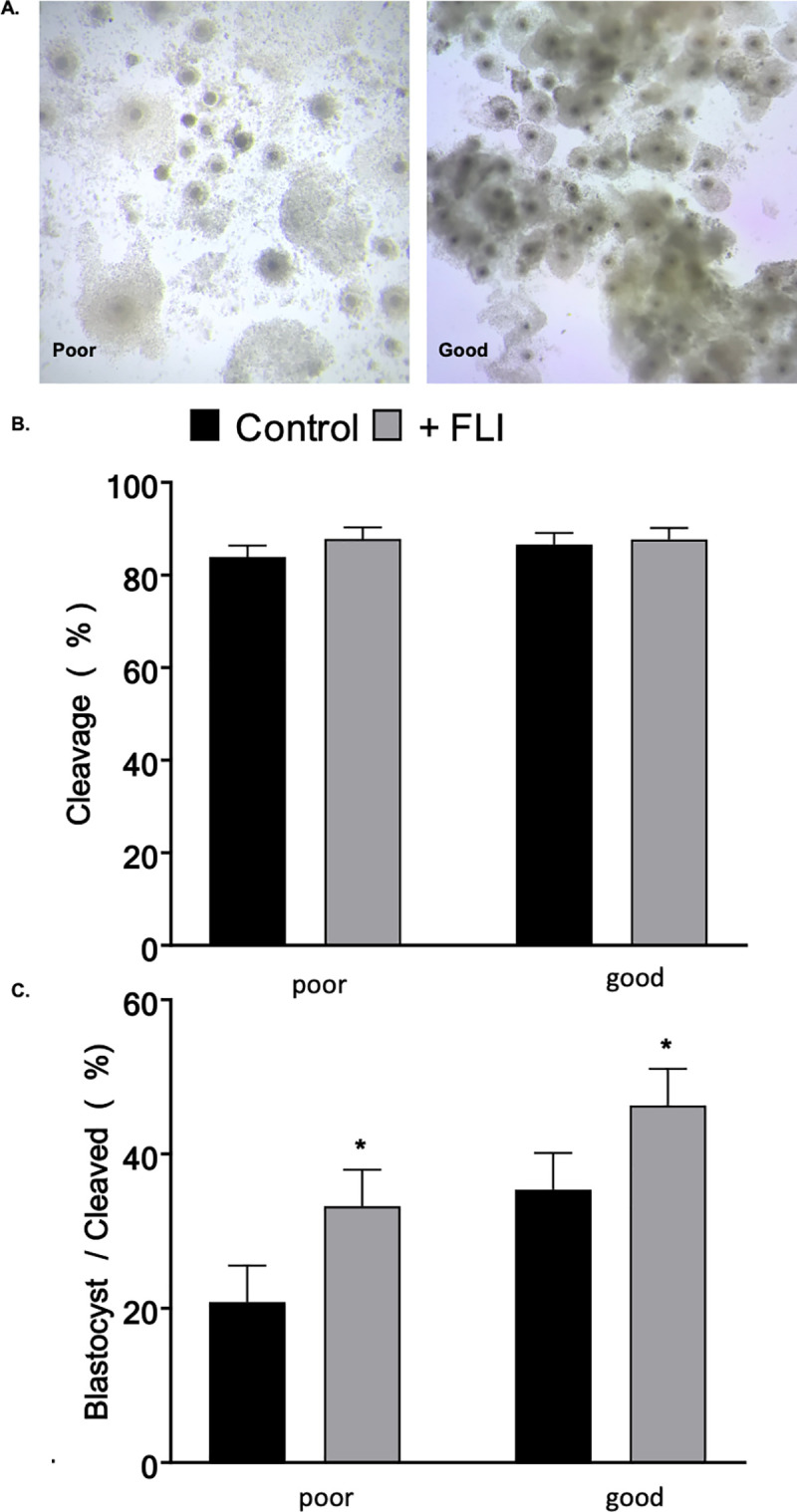
FLI Supplementation to culture medium improves embryo development. Bovine oocytes (n = 1,066) were of different qualities. (A) Example of oocytes classified as poor quality (470) or good quality (596) (B) Proportion of zygotes that underwent at least one cellular division. (C) The extent of the effect of FLI supplementation in culture medium on development to blastocyst stage varies based on oocyte quality/source. Asterisk (*) indicates statistical differences (*P <* 0.05). Values are LSMEANS ± SEM.

Abattoir derived COCs (n = 4,400 across 16 replicates) were received in oocyte maturation medium. COCs were received from two different companies that provide abattoir derived COCs. From both sources, COCs were placed in equilibrated OMM (50 COCs/tube) overlaid with oil and shipped via portable incubator set to 38.5˚C and left in tubes until fertilization. For 6 replicates, oocytes were assessed upon receiving and classified as poor quality or good quality (Fig 3A). Poor quality COCs (n = 470) had few expanded cumulus cells, dark and granulated ooplasm, or appeared very pale. Good quality COCs (n = 596) had multiple layers of cumulus cells, and ooplasm that was uniform and dark in color. Given the apparent variation in oocyte quality, the remaining 10 replicates only used COCs from company 2 (consistent cumulus cell expansion and homogenous ooplasm).

Fertilization and embryo culture were performed as described for previous experiments. For culture, 50 putative zygotes were placed in a 4-well dish containing 500 μl of SOF-BE2 topped with 300 μl mineral oil. Half of the putative zygotes were placed in culture medium supplemented with FLI. Embryos were cultured in the same environment as previously described. Embryos cultured with or without FLI were subjected to cryopreservation (n = 117; 51 control, 66 +FLI, across 5 replicates), thawed, and those that re-expanded were subjected to TUNEL (n = 79; 31 control, 48 +FLI) or determination of lipid content (n = 162; 72 control, 90 +FLI) as described above.

#### Cell number

The number of nuclei in the trophectoderm and inner cell mass was determined after immunolocalization of the trophectoderm marker CDX2 and a nuclear stain (Hoescht) in blastocyst (6–1) stage embryos. Day 7 or day 8 embryos were collected, washed with PBS-PVP and fixed for 20 min in 4% paraformaldehyde. Fixed embryos were permeabilized as previously described, washed, and incubated in blocking buffer [5% BSA (w/v)] for one h at room temperature. Embryos were incubated in the primary antibody [anti-CDX2 (mouse monoclonal antibody) BioGenex Fremont, CA, USA] in the dark at 4˚C overnight. Embryos were washed and placed into the secondary antibody (mouse anti-goat, Alexa 488) for one h at room temperature. Embryos were washed with PBS-PVP three times and nuclei were stained with Hoechst 33342 (1 μg/ml dilution) for 20 min in the dark. Each replicate contained a secondary antibody control embryo that was subjected to the above method minus incubation in the primary antibody (S1 Fig). Samples were mounted on slides in Slow fade® Gold antifade reagent (Thermo Fisher Scientific) and analyzed by fluorescent microscopy. Excitation/emission was 350/461 for Hoechst 33342 and 485/535 for Alexa 488. The number of trophectoderm nuclei was subtracted from the total number of nuclei to determine the inner cell mass number. Embryos (n = 160; 72 control and 88 +FLI) were analyzed across three replicates.

#### Classification of cytoskeleton integrity

**Fig 4:**
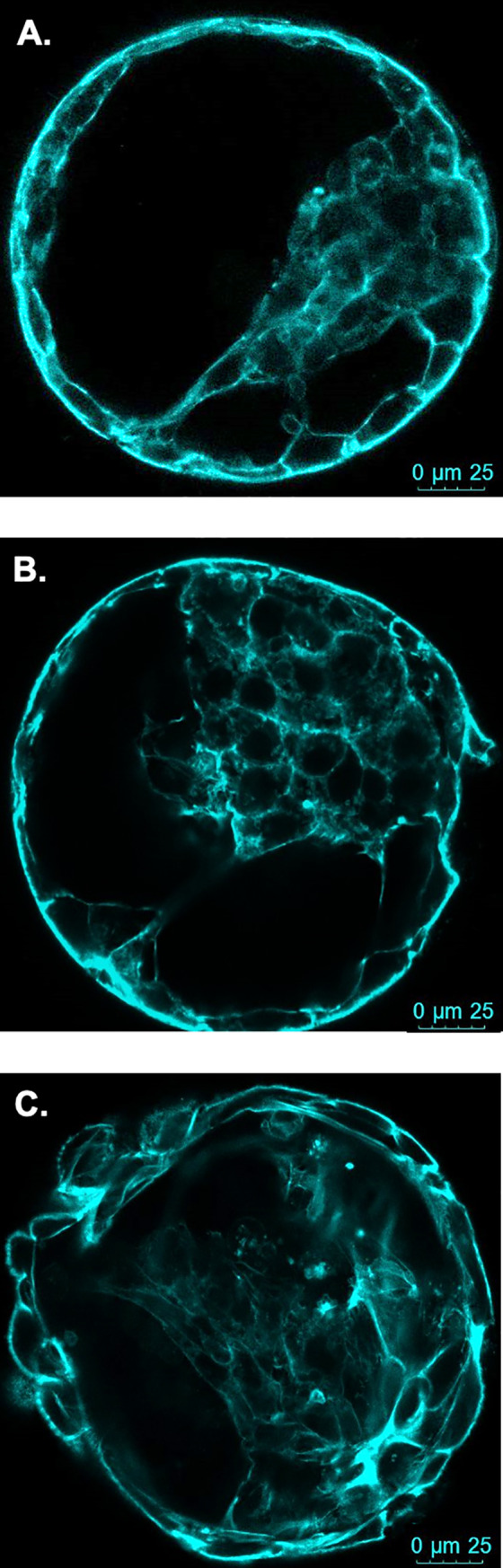
Representative images of bovine blastocysts showing the actin filament staining. (A) Grade 1 blastocyst stage embryos displayed sharp staining of actin filaments and cells are clearly organized as inner cell mass or trophectoderm. (B) Grade 2 shows less distinct outlining of the cells and some breaks in the cytoskeleton structure. (C) Grade 3 is characterized by large gaps in actin staining and some actin within the cytoplasm. Actin filaments were visualized in 101 fresh (n = 52 control, n = 49 +FLI) and 52 (n = 22 control, 30 +FLI) thawed embryos.

Blastocyst stage embryos were collected fresh at day 7 or at 24 h post thaw. A 24 h thawing period allowed for most of the embryos to re-expand but not to the point of hatching from the zona pellucida. Blastocysts were washed in PBS-PVP and fixed for 20 min in 4% paraformaldehyde. After permeabilization, they were washed three times with PBS-PVP, and placed in rhodamine phalloidin (Cytoskeleton Inc.) at 0.04 μg/ml. Incubation proceeded in the dark for 45 min at room temperature. Blastocysts were washed three times with PBS-PVP and incubated in Hoechst 33342 staining (1 μg/ml) for 20 min in the dark. Finally, blastocysts were mounted on slides in Slow fade® Gold antifade reagent (Thermo Fisher Scientific) and analyzed by using confocal microscopy (Leica TCS SP8 MP). Excitation/emission was 350/461 for Hoechst 33342 and 540/585 for rhodamine phalloidin. ImageJ Software 1.46r (National Institutes of Health) was used to determine the number of disruptions (breaks) in the cytoskeleton of the cells and embryos were graded as previously described [20, 21] (Fig 4). Fresh (non-cryopreserved embryos: n = 101, 52 control and 49 +FLI) and thawed (n = 52, 22 control and 30 +FLI) embryos were analyzed across three replicates.

#### Detection of reactive oxygen species (ROS)

CellROX®Green (Thermo Fisher Scientific) was used to measure oxidative stress [41]. Briefly, day 7 fresh or 24 h post-thaw blastocysts were transferred to fresh SOF-BE2 containing CellROX®Green (10 mM) and cultured at 38.5°C in a humidified atmosphere of 5% (v/v) O_2_ and 5% (v/v) CO_2_ with the balance N_2_ for 30 min. Embryos were collected, washed with PBS-PVP, and fixed for 20 min in 4% paraformaldehyde. Nuclei were stained with Hoechst 33342 (1 μg/ml dilution) for 20 min in the dark. Samples were mounted on slides in Slow fade® Gold antifade reagent (Thermo Fisher Scientific) and analyzed by using fluorescence microscopy. Excitation/emission was 350/461 for Hoechst 33342 and 485/520 for CellROX®Green. ImageJ Software 1.46r (National Institutes of Health) was used to quantify the mean intensity units per embryo to estimate ROS levels. A total of 99 fresh embryos and 70 cryopreserved embryos were analyzed across 3 replicates.

#### Statistical analysis

All analyses were conducted using SAS (V 9.4, SAS Institute, Cary, NC). Differences in transzonal projection dissociation were analyzed by analysis of variance using PROC GLM. The model included treatment, hour and the interaction of treatment and hour. Maturation rate was analyzed using PROC GLIMMIX. The model included treatment as a fixed factor and replicate as a random factor. A binomial regression model was used. Development, lipid content, and apoptosis (in both FLI added to OMM and FLI added to culture medium) were analyzed by analysis of variance using PROC GLM. The model included replicate, treatment, and the interaction of replicate and treatment. In the analysis for development for FLI added to culture medium, company was included as a covariate. Differences in cell number and cell ratio were analyzed by analysis of variance using PROC GLM. The model included replicate, treatment, and the interaction of replicate and treatment. Embryo hatching following cryopreservation and reactive oxygen species were analyzed by analysis of variance using PROC GLM. The model included replicate and treatment. Differences in cell membrane integrity (pre and post thaw) were analyzed using PROC GLIMMIX. The model included treatment and replicate as well as the interaction of the two. A binomial regression model was included. In all analyses significance was determined as *P* < 0.05 and results are shown as least square means ± standard error of the mean.

## Results

### Effects of FLI supplementation in oocyte maturation medium

#### Transzonal projection dissociation, oocyte maturation, and embryo development

**Fig 5:**
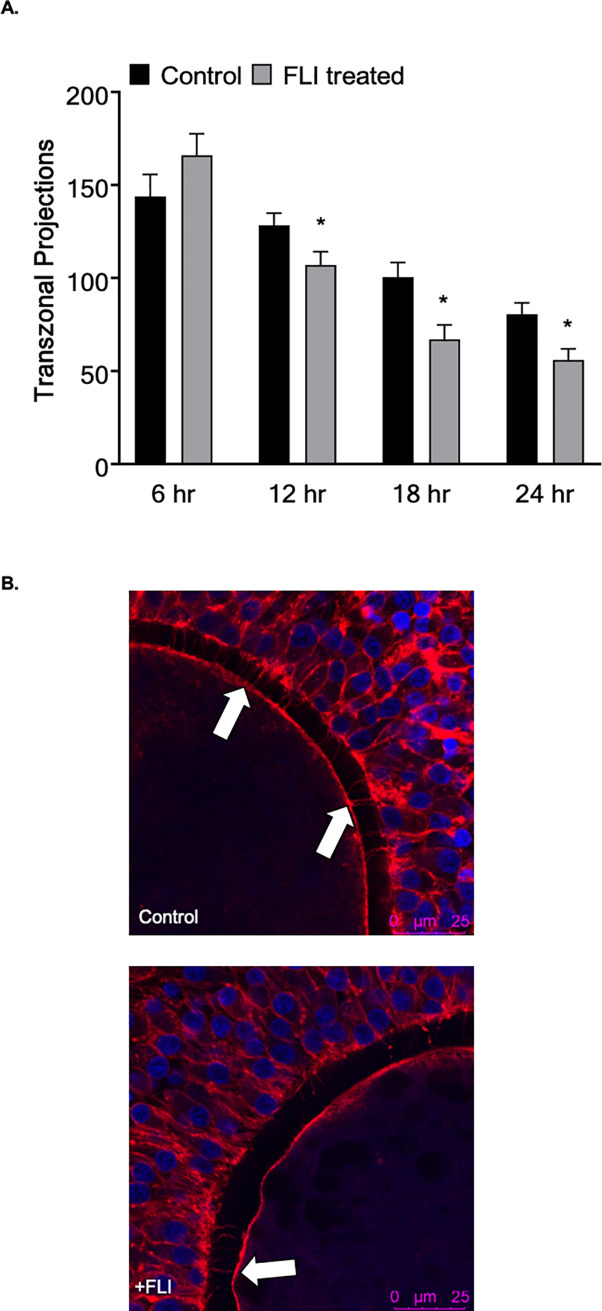
FLI supplementation advances transzonal projection dissociation. (A) The effect of the addition of FLI to oocyte maturation medium on the number of TZP at 6, 12, 18, and 24hr. (n = 198; 100 control and 98 +FLI). (B) The images are representative of TZP dissociation at 24hr of maturation. Arrows indicate intact TZP. Asterisk (*) indicates statistical differences (*P <* 0.05).

Supplementation of FLI to our current OMM was investigated to understand the effects on oocyte maturation and preimplantation embryo development in vitro. The addition of FLI to OMM had no effect (*P >* 0.05) on the number of transzonal projections at 6 h between FLI treated and control groups, (166.3 ± 8.6 vs 143.9 ± 8.8), respectively. However, there was a significant treatment by time interaction as FLI-treated COCs had fewer (*P <* 0.05) transzonal projections at 12 h, 18 h, and 24 h compared to the controls (Fig 5A). Additionally, supplementing FLI to OMM resulted in an increase (*P <* 0.05) in the percentage of oocytes that reached metaphase II (Table 1). However, there was no difference in cleavage or blastocyst percent between treatments (Table 1).

**Table 1:** In vitro development of bovine embryos supplemented with and without FLI during maturation.

Group^1^	n	MII oocytes (%)^2^	Cleavage (%)	Blastocyst (%)	Blastocyst/Cleaved (%)
Control	1495	62.6 ± 0.40^a^	80.0 ± 0.73	34.2 ± 1.07	42.9 ± 1.35
FLI	1495	75.6 ± 0.34^b^	80.3 ± 0.73	35.4 ± 1.07	44.0 ± 1.35

^a,b^Numbers with different superscripts differ (*P<0*.*05*).

^1^All results are reported as least square means ± standard error of the mean.

^2^An analysis from a subset of oocytes (n = 304) collected from the total.

#### Lipid content and cryo-survival

**Fig 6:**
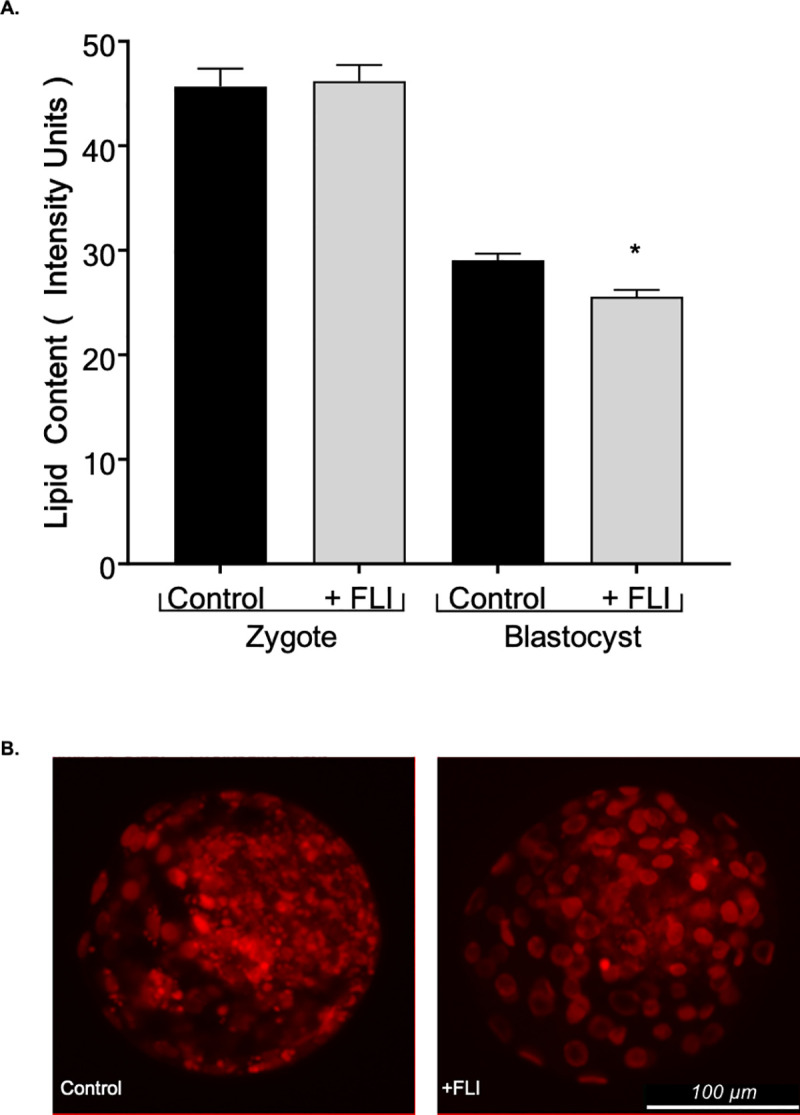
FLI supplementation during oocyte maturation decreases lipid content at the blastocyst stage. (A) Lipid content was compared at the zygote (n = 239; 109 controls and 120 +FLI) and blastocyst stage (n = 222; 116 controls and 106 +FLI). (B) Blastocyst stage bovine embryos illustrating Nile red staining by fluorescence microscopy used to determine lipid content. Asterisk (*) indicates statistical differences (*P <* 0.05).

Nile Red staining was used to quantify intracellular lipid content in zygotes and blastocyst stage embryos. The addition of FLI into the maturation medium had no effect (*P >* 0.05) on the lipid content at the zygote stage between the two treatments (Fig 6A). Interestingly, the embryos from the FLI supplemented group had decreased lipid content at the blastocyst stage compared to the controls (*P <* 0.001, Fig 6A). Supplementation of maturation medium with FLI, did not improve the ability of the embryo to withstand cryopreservation between the treatments (*P >* 0.05, Table 2) when survivability is measured by hatching rate in vitro. Likewise, there was no difference in DNA fragmentation between the two treatments (*P >* 0.05).

**Table 2:** Hatching rates post-thaw of bovine embryos supplemented with and without FLI during maturation following cryopreservation by slow-freezing.

		Hatching (%)^1^		
Group	n	24h	48h	72h
Control	51	36.2 ± 0.05	58.8 ± 0.07	67.0 ± 0.07
FLI	66	27.1 ± 0.05	60.6 ± 0.07	63.3 ± 0.07

^1^Results reported as least square means ± standard error.

### Effects of FLI supplementation in embryo culture medium

#### Embryo development

The aim of this experiment was to analyze the effect of FLI on embryo culture. Supplementation of SOF-BE2 with FLI had no effect (*P >* 0.05) on cleavage percentage between the control and FLI treated groups (Table 3). However, the addition of FLI to the culture medium resulted in an increased (*P <* 0.0001) proportion of embryos that developed to the blastocyst stage (Table 3). Oocytes of variable quality were used for IVP during this study. To understand the effect of FLI supplementation on oocytes of different qualities, oocytes were assessed upon receiving and classified as poor quality or good quality (Fig 3A). The extent of the effect of FLI supplementation in culture medium on development to the blastocyst stage varied based on oocyte quality/source (Fig 3C). In both cases those treated with FLI had an increase (*P < 0*.*05*) in development to the blastocyst stage.

**Table 3:** In vitro development and number of nuclei in bovine embryos supplemented with and without FLI in culture.

					Cell Number^3^		
Group^1^	n	Cleavage (%)^2^	Blastocyst (%)	Blastocyst / Cleaved (%)	TE^4^	ICM^5^	Total
Control	2104	82.4 ± 0.6	27.2 ± 1.1^a^	32.9 ± 1.3^a^	73.0 ± 2.7	66.3 ± 2.4	140.2 ± 4.3
FLI	1930	83.7 ± 0.6	38.6 ± 1.1^b^	46.2 ± 1.3^b^	68.0 ± 2.7	64.9 ± 2.4	133.2 ± 4.3

^a,b^Numbers with different superscripts differ (P<0.05).

^1^Values are presented as least square means ± standard error.

^2^Embryos that underwent at least one cellular division.

^3^A subset (160) of embryos was used for cell number analysis.

^4^Trophectoderm cells.

^5^Inner cell mass (equal to total nuclei minus trophectoderm).

#### Cell number, lipid content, oxidative stress, cytoskeleton integrity

The objective of this experiment was to identify quality parameters affected when zygotes are cultured in the presence of FLI. Embryo cell number, lipid content, oxidative stress, and cytoskeleton integrity were assessed at the blastocyst stage. The supplementation of FLI to culture medium had no effect (*P >* 0.05) on the number of trophectoderm, inner cell mass, or total cells (Table 3) compared to the control group. Likewise, there was no effect (*P >* 0.05) on the ratio of trophectoderm to inner cell mass nuclear number (1.35 ± 0.06 control vs 1.24 ± 0.05 +FLI). Lipid content in the control group (42.8 ± 1.5 mean intensity units) was not different (*P >* 0.05) compared to the FLI treatment (44.5 ± 1.6 mean intensity units). In addition, no difference (*P >* 0.05) was found in the presence of reactive oxygen species, measured by CellROX®Green, between the control and FLI treated groups (56.98 ± 4.93 vs 56.09 ± 4.13 mean intensity units), respectively.

To determine the integrity of the cytoskeleton, blastocyst stage embryos were stained with rhodamine phalloidin, imaged via confocal microscopy, and graded (Fig 4). A greater (*P <* 0.05) proportion of FLI-treated embryos were quality grade 1 (15.9% ± 4.07 vs 39.2% ± 4.07), indicating that when cultured in the presence of FLI, embryos have a higher quality cytoskeleton structure and cell organization.

#### Cryo-survival

**Fig 7:**
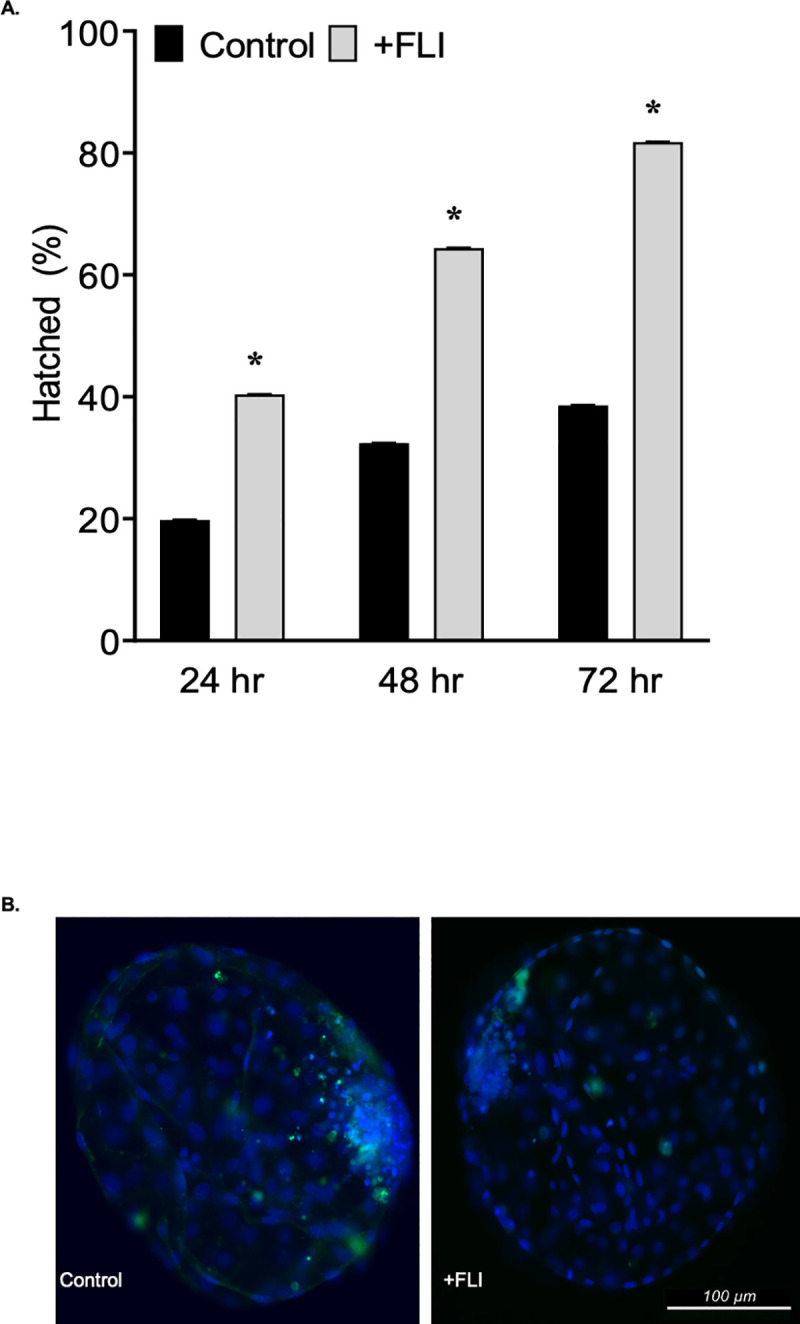
FLI supplementation during embryo culture improves cryopreservation. (A) The effect of the addition of FLI to culture medium and post-thaw hatching rate (n = 117; 51 control and 66 +FLI). (B) Images of bovine embryos post-thaw subjected to the TUNEL assay (green) and fluorescence imaging. Asterisk (*) indicates statistical differences (*P <* 0.05).

Blastocyst stage embryos [quality grade 1 and stage 6 or 7 [45] were slow frozen and hatching from the zona pellucida post-thaw was recorded as an indicator of survivability. Embryos cultured in the presence of FLI had an increased (*P <* 0.05) ability to withstand cryopreservation by slow-freezing. At 24 h post thawing the percentage of FLI treated embryos (40.4% ± 0.04) that had hatched from the zona pellucida was double that of the control group (19.8% ± 0.04). This difference was maintained at 48 and 72 h (Fig 7A). A TUNEL assay indicated that embryos supplemented with FLI have a decreased (*P <* 0.05) proportion (6.70% ± 1.32 vs 18.06% ± 1.62) of apoptotic cells compared to control embryos (Fig 7B). For the control and the FLI treated groups, the average total cell numbers were 149.89 ± 22.20 and 199.03 ± 18.10, respectively. The average number of apoptotic cells was 23.04 ± 4.78 for the control group and 9.52 ± 3.90 for the FLI treated group. Reactive oxygen species and cytoskeleton integrity were also determined following cryopreservation. The cytoskeleton integrity of embryos supplemented with FLI was not different (*P >* 0.05) compared to the control embryos (42.2% ± 4.77 +FLI vs 20.2% ± 4.77 controls). Likewise, there was no difference (*P >* 0.05) in reactive oxygen species between the FLI (40.9 mean intensity units ± 2.59) and control (33.8 mean intensity units ± 3.16) groups.

## Discussion

Supplementation of the cytokine combination FGF2, LIF, and IGF1 (FLI) has been shown to increase the efficiency of in vitro porcine embryo production in [37]. These cytokines have been used individually in in vitro bovine embryo production systems and yielded positive results [27, 35, 51–53], so it is possible that combining these cytokines has the potential to increase bovine embryo development in vitro. Thus, the objective of this study was to investigate the role of FLI in the in vitro bovine embryo production system and evaluate the phenotypic effects on the oocyte and embryo.

The supplementation of FLI into maturation medium resulted in an earlier dissociation of the transzonal projections from the cumulus cell to the oocyte. Transzonal projections have a role in lipid accumulation within the embryo [12]. Our results support this finding as blastocyst stage embryos that developed from the oocytes supplemented with FLI had reduced lipid accumulation compared to those that did not receive FLI supplementation. It is possible that the transzonal projections act as an entryway for lipids or lipid precursors to enter the oocyte. Therefore, the earlier dissociation of the entry mechanism may prevent these molecules from traversing into the oocyte. Furthermore, it has been shown that lipid content decreases around the time of fertilization and the first cleavage division, then increases following the maternal to zygotic transition [54]. Suboptimal maturation conditions may result in excessive lipid uptake that is accumulated instead of used for energy. It is possible that FLI supplementation provides the maturation medium with a necessary component for regulation of lipid synthesis and accumulation. Growth factors such as LIF, IGF1, and FGF2 activate the mitogen‐activated protein kinase (MAPK) signaling cascade [51, 55, 56], which has a role in cellular metabolism and lipolysis [57–59].

One explanation is that the cytokines that compose FLI are interacting with lipid modulators that have a role in lipid droplet lipolysis and mobilization of energy stores. Interestingly, the decreased lipid content at the blastocyst stage did not result in an increased survivability or reduced DNA fragmentation following cryopreservation. This differs from previous studies showing that decreased lipid content resulted in increased survival after cryopreservation [60, 61]. Nile red staining was used to quantify the intracellular lipid content within the embryo. This method tags neutral lipids, specifically, triglycerides, the most abundant lipid in oocytes and embryos [54]. Triglycerides have been detected in higher amounts in in vitro derived embryos compared to in vivo [62]. Our results indicate that lipid content, or neutral lipids rather, may not be the factor that determines survival through cryopreservation, and further research is needed to understand the quality parameters that are important for survival of embryos to slow-freezing.

An increase in lipid content was observed at the blastocyst stage in the control group compared to the FLI supplemented but not in the zygote stage embryos. It is possible that the addition of FLI during maturation results in changes in lipid metabolism regulation that carry through to the blastocyst stage. There is evidence that embryos accumulate lipids from their culture environment [63]. At the zygote stage, the embryo has had minor exposure to the culture environment, but by the blastocyst stage the embryo has been exposed to the medium for 7 days. During this time, embryos with dysregulated lipid metabolism could accumulate excess lipids. We have shown that the addition of FLI to the maturation medium affects the regulation of lipid metabolism in blastocyst stage embryos as evidenced by a decrease in lipid content at this stage.

The addition of FLI during maturation did increase the proportion of oocytes that reached the metaphase II stage of meiosis. After removal from the ovary, timing of resumption and progression of meiosis depend largely on the maturation conditions [64]. Specifically, leukemia inhibitory factor (LIF), has been shown to increase both cytoplasmic and nuclear maturation when supplemented to in vitro oocyte maturation medium [11]. Oocyte maturation has a key role in determining the developmental competence of the oocyte to reach the blastocyst stage [6]. Interestingly, in this study, the increase in oocytes reaching the metaphase II stage did not result in an increase in the proportion of putative zygotes that underwent at least one cleavage division or reached the blastocyst stage. This suggests a role of FLI in driving the progression to metaphase II during nuclear maturation, however, the role of FLI during cytoplasmic maturation remains to be elucidated. In both groups, the percentage of oocytes that reached metaphase II by twenty-four hours of maturation was lower than the cleavage rate. One explanation would be that oocytes with competence to cleave had not reached metaphase 2 by twenty-four hours. Aspiration of the COC from the ovary did not occur in house so the size and maturity of the follicle is unknown. Some of the COCs may have finished maturation to metaphase 2 during fertilization and therefore had the capacity to cleave. In addition, some of them could be parthenotes, as is common in IVP. It should be noted that the current oocyte maturation medium used in this system contains serum with undefined growth factors. We recognize that the undefined molecules present in the serum may be masking the effect of FLI and that this combination of cytokines may be increasingly beneficial in a serum free system.

The addition of FLI at the beginning of culture increased embryonic development to the blastocyst stage. When compared to the US average (25.0%) [45] this is an increase of over ten percentage points. This is consistent with other work that has shown that these cytokines individually, or combinations of these cytokines can increase embryo development in vitro [27, 35, 51–53], indicating that these molecules may be stimulating key regulatory pathways that are important for embryo development.

In this study we observed a difference in cytoskeleton integrity between embryos treated with and without FLI. Fewer disruptions in the cell cytoskeleton and formation of a well-defined inner cell mass and blastocoel within the embryo would indicate that these embryos may be more prepared to undergo stressful processes such as embryo transfer or cryopreservation. While little is known about these cytokines and cell allocation, it is possible that FLI is stimulating pathways involved in cell-to-cell junction formation, cell organization, and compaction. FGFs acting through FGFR have been shown to have a role in cell differentiation, migration, and regeneration [65]. In mice, IGF-1 receptor activation is linked with trophectoderm cell survival and E-Cadherins, molecules involved in cell clustering [66]. Both of these cytokines work through activation of both the MAPK signaling cascade and PI3K pathways which are known to be involved in cell division and self-renewal.

Cryopreserving bovine embryos is an important component when it comes to the adoption and use of IVP. Supplementing FLI at beginning of culture increases the survivability of embryos following slow freezing, and decreased DNA fragmentation post-thawing. Previous studies have indicated that LIF supplementation prior to cryopreservation resulted in an increase in the post thaw survivability of embryos [30]. Additionally, it has been shown that IGF-1 alone or when combined with LIF increases the number of total cells and trophectoderm cells within the embryo following freezing [34]. In this study, lipid content was not a determining factor in post-thaw embryo survival. We have shown a role of FLI in improving post-thaw embryo survival in vitro but further studies are needed to understand if these results translate into improved pregnancy outcomes for IVP and frozen embryos.

The effects of FLI were not expressed through a difference in nuclear number, lipid content, or oxidative stress, indicating that the effects of FLI on quality are not reflected in the blastocyst stage embryo, but rather FLI is affecting the embryo earlier in development, which remains to be elucidated. Recent research has shown that Bovine Serum Albumin (BSA) interferes with the fibroblast growth factor signaling cascade [67]. With BSA present in the current culture medium, it is possible that it is blocking exogenous FGF2 from exerting its effect. The pathway associated with lineage commitment has been altered in mouse blastocyst stage embryos when they were cultured in the presence of BSA [67], thus, the same could be true for bovine.

In summary, the supplementation of FLI during the in vitro production of bovine embryos proves to be beneficial by improving preimplantation embryonic development and embryo quality. The positive influence of FLI is evident in oocyte maturation and embryo lipid content when added to the maturation medium, as well as embryo development and survivability when added to the culture medium. Further research will help to elucidate the mechanisms through which FLI modulates embryo development, quality, and survivability.

## Supporting information

S1 Fig Secondary antibody control. Secondary antibody control for localization of CDX2. Embryos were prepared as described above for determination of cell number with the primary antibody step omitted.
(TIF)
