# Supplementary figures and images for: Improved cryopreservation of in vitro produced bovine embryos using FGF2, LIF, and IGF1
Source: PLoS One. 2021 Feb 3;16(2):e0243727. doi: 10.1371/journal.pone.0243727 (PMC7857633; doi:10.1371/journal.pone.0243727)

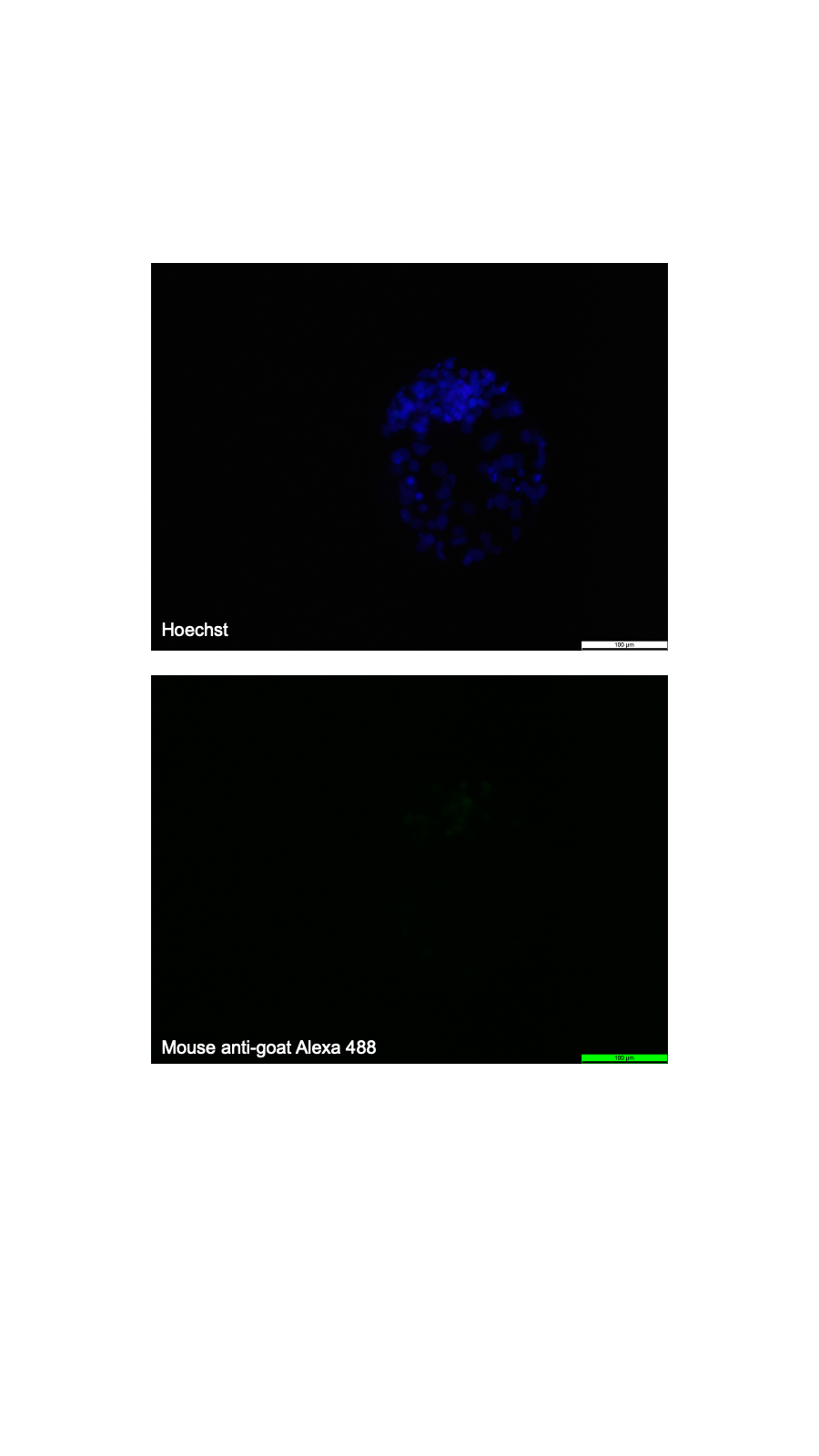

Supplement: S1 Fig — Secondary antibody control for localization of CDX2. Embryos were prepared as described above for determination of cell number with the primary antibody step omitted. (TIF) [file pone.0243727.s001.tif]
